# Passive temperature control based on a phase change metasurface

**DOI:** 10.1038/s41598-018-26150-9

**Published:** 2018-05-16

**Authors:** Sheng-Rui Wu, Kuan-Lin Lai, Chih-Ming Wang

**Affiliations:** Department of Opto-electronic Engineering, National Dong Hwa University, Hualien, 97401 Taiwan

## Abstract

In this paper, a tunable mid-infrared metasurface based on VO_2_ phase change material is proposed for temperature control. The proposed structure consisting of a VO_2_/SiO_2_/VO_2_ cavity supports a thermally switchable Fabry-Perot-like resonance mode at the transparency window of the atmosphere. Theoretically, the radiative cooling power density of the proposed metasurface can be switched to four-fold as the device temperature is below/above the phase change temperature of VO_2_. Besides radiative cooling, a passive temperature control application based on this huge cooling power switching ability is theoretically demonstrated. We believe the proposed device can be applied for small radiative cooling and temperature control applications.

## Introduction

Plasmonic metamaterials and metasurfaces have been demonstrated for their ability to manipulate almost the entire range of properties of incident electromagnetic waves, for example, manipulating the amplitude, polarization, propagation direction, frequency and phase and so on^1^. A lot of corresponding applications have been proposed based on the metamaterials and metasurfaces. Recently, manipulating thermal emission by using plasmonic metamaterials has received great attention and various applications are thus proposed^2,3^. A series of works shows that the thermal emission from plasmonic metamaterial can be modified according to the geometric parameters of the structure^4,5^. As the emission wavelength is tuned to be at the transparency window of the atmosphere, cooling effects based radiation can be achieved. In 1977, Bartoli *et al*. first demonstrated passive radiative cooling at night^6^. Daytime radiative cooling is more challenging compared to nighttime radiative cooling, however, owing to the solar heating. In 2014, Raman *et al*. first successfully demonstrated daytime passive cooling^7^. In that study, a 5 °C reduction below the ambient temperature under direct sunlight was experimentally demonstrated. Since then, radiative cooling, i.e. radiating heat to the cold sink of outer space via the transparency window of the atmosphere, has received a lot of research interest. Additional energy and resources to carry heat away are not needed during the radiative cooling process. Alongside these experiments, a lot of theoretical works aimed at designing various photonic and plasmonic structures for radiative cooling applications have also been proposed^8–11^. In particular, low-cost radiative cooling without external active devices is of much interest.

By combining phase change material and metamaterial, a tunable/reconfigurable metamaterial can be realized. For example, the focused features of a germanium-antimony-tellurium (GST) alloy metalens can be optically controlled and switched^12^. The resonant frequency of a metamaterial can be thermally tuned based on VO_2_ phase transition^13^. This means one can not only tune the emission peak at the transparency window of the atmosphere by tuning the geometric parameters of the metamaterial but also by tuning the temperature of a phase change material. Besides of reconfigurable metamaterials, VO_2_ has been demonstrated in various applications such as thermal diodes^14^, thermal transistors^15^, thermal memories^16^ and thermochromic smart coating^17^.

In this paper, we propose a SiO_2_/VO_2_ multilayer metasurface for a radiative cooling device. The SiO_2_/VO_2_ multilayer forms a thermally tunable metal/insulator/metal (MIM) cavity. The absorption spectrum of the designed structure shows the absorption at the transparency window of the atmosphere regime dramatically changed as the insulator phase VO_2_ phase changes to be a metal phase one. We believe the proposed structure can be used as a tunable radiative cooling and passive temperature control device.

## Schematic of the Phase Change Metasurface

**Figure 1:**
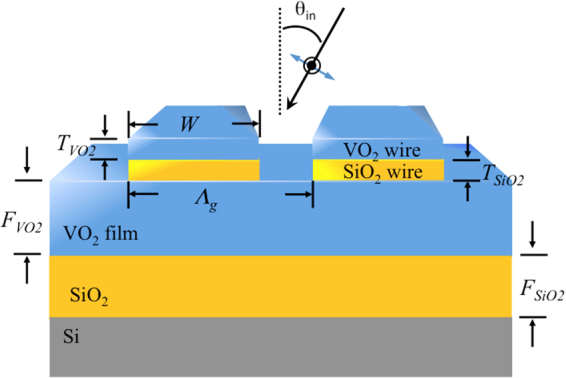
Side-view diagram of the investigated phase change metasurface. The thickness of the SiO_2_ film and VO_2_ film is denoted by F_SiO2_ and F_VO2_, respectively. The thickness of the SiO_2_ wire and VO_2_ wire is denoted by T_SiO2_ and T_VO2_, respectively. The width of the SiO_2_ wire and VO_2_ wire are identical and denoted by W. The periodicity of the structure is denoted by Λ_g_. The incident angle is denoted by θ_in_.

The schematic of the investigated phase change metasurface is shown in Fig. 1. The structure consists of a silicon substrate, SiO_2_ film, VO_2_ film, SiO_2_ wire and VO_2_ wire, from bottom to top. The thickness of the SiO_2_ film and VO_2_ film is denoted by F_SiO2_ and F_VO2_, respectively. The thickness of the SiO_2_ wire and VO_2_ wire is denoted by T_SiO2_ and T_VO2_, respectively. The width of the SiO_2_ wire and VO_2_ wire are identical and denoted by W. The incident angle is denoted by θ_in_. The periodicity of the structure is denoted by Λ_g_. Here, Λ_g_ is fixed to be 3 μm. This structure can be fabricated by sequentially coating SiO_2_/VO_2_/SiO_2_/VO_2_ film onto a silicon substrate. Then, the SiO_2_ wire and VO_2_ wire can be made by etching the top SiO_2_/VO_2_ film using a one-step focused ion beam (FIB).

The optical properties of the phase change metasurface are simulated using the rigorous coupled-wave analysis (RCWA) method. The complex dielectric constants of SiO_2_ are taken from ref.^18^. The complex optical constants of the monoclinic insulator phase and rutile metal phase of VO_2_ phase change material are taken from ref.^19^. Although the phase change temperature of VO_2_ might be slightly changed due to the difference in microstructure and crystallinity at different nucleation conditions resulting from various fabrication processes, the most common phase change temperature is 68 °C. It has also been shown that the transition temperature of the insulator-to-metal transition of VO_2_ is narrower than 1 °C^20^. Therefore, we can simply assume that the VO_2_ is in metal-phase as the device temperature T_dev_ > 68 °C while it is in insulator-phase as T_dev_ < 68 °C.

## Spectral Response of Proposed Phase Change Metasurface

**Figure 2:**
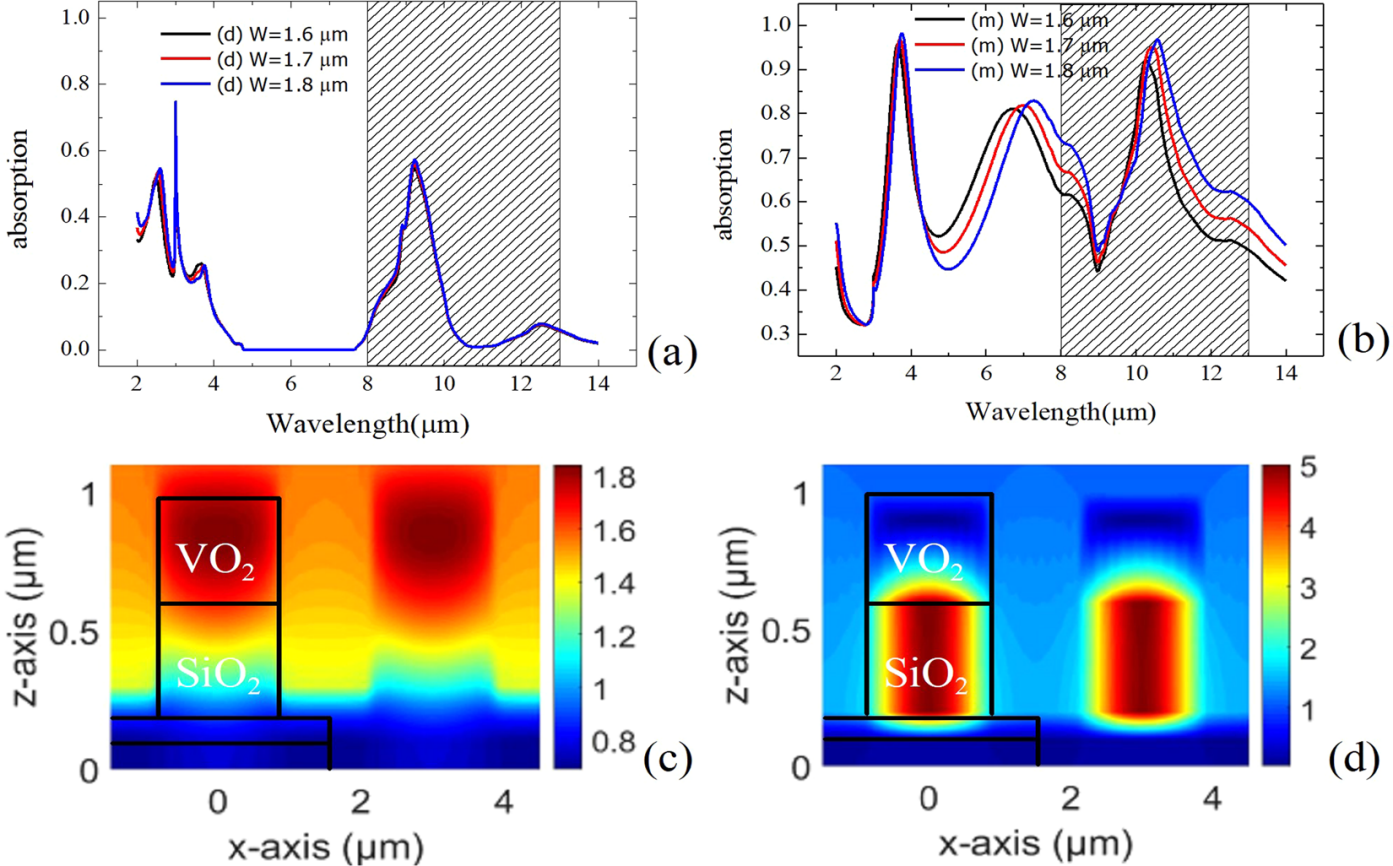
Absorption spectrum of the VO2 metasurface as (**a**) Tdev < 68 °C and (**b**) Tdev > 68 °C. The shaded area highlights the region of the transparency window of the atmosphere. Hy field distribution of the VO_2_ metamaterial as (**c**) T_dev_ < 68 °C and (**d**) T_dev_ > 68 °C at resonant wavelength.

Figures 2a,b shows the absorption spectra of the VO_2_ metasurfaces for a normally incident light. The incident light is TM-polarized (electric vector is perpendicular to the grating grooves). The TE-polarized light is also simulated. However, because of no significant resonance in the investigated spectral range, it is not shown here. Both the insulator-phase and metal-phase of VO_2_ are simulated and shown in Fig. 2a and b, respectively. Here, the simulated geometric parameters of the VO_2_ metasurfaces are F_SiO2_ = 100 nm, F_VO2_ = 100 nm, T_SiO2_ = 400 nm, T_VO2_ = 400 nm and Λ_g_ = 3000 nm. Black, red, and blue solid lines represent the metasurfaces with W = 1.6 μm, W = 1.7 μm and W = 1.8 μm, respectively. For λ = 8 μm to 13 μm, it is referring to the transparency window of the atmosphere. For the insulator-phase of VO_2_, the spectral response of the metasurface shows a clear absorption peak at 9.6 μm with an absorption of 0.6. This absorption is mainly contributed by the longitudinal optical (LO) vibration mode of SiO_2_. Additionally, it has also reported that the LO/TO (transverse optical) vibration frequency of deposited SiO_2_ depends on the film thickness^21^. In our simulation, we cannot faithfully represent characteristics of the thickness-dependent vibration frequency. Owing to the fact that the absorption is mainly contributed by the inherent absorption of SiO_2_, it is shown that the resonance wavelength is almost the same as W increases from 1.6 μm to 1.8 μm.

As the device temperature T_dev_ > 68 °C, it can be seen that the absorption of the metasurface dramatically increases. The absorption at 9.6 μm is 0.6, which is similar to that of the metasurface of the insulator-phase VO_2_. We also find a resonance absorption peak at 10.5 μm for W = 1.6 μm. As W is increasing, the resonance wavelength shows a redshift that is almost linearly with a slope of 1.5 wavelength/width.

From the localized field distribution at absorption peaks, we can observe an anti-node of standing wave resonance along the z-direction at the SiO_2_ wire for the metasurface of the insulator-phase VO_2_ as shown in Fig. 2c. The magnitude of standing wave is 1.8 fold compared to that of the incident field. In contrast, the Hy field (y-component of the magnetic field) is localized at the VO_2_/SiO_2_/VO_2_ cavity, as shown in Fig. 2d. The metallic phase of VO_2_ forms a metal/insulator/metal cavity to support a magnetic resonance mode. From the mode pattern, we can recognize that the resonance mode is a fundamental Fabry-Perot mode. The magnetic field oscillates along the x-axis. Consequently, a wild W corresponds to a longer resonance cavity that leads to a redshift of resonance as already shown in Fig. 2(b). This magnetic resonance mode prolongs the absorption path of the SiO_2_. As a result, the SiO_2_ absorption corresponds to the TO vibration around 10μm and bond-bending vibration around 11 μm to 13μm can be enhanced. The absorption enhancement can be switched off as the device temperature is below the phase change temperature of VO_2_.

## Radiative Cooling Based on VO_2_ Metasurface

Here, we assume that the heat transfer is based on thermal radiation only. The heat transfer via convection and conduction is ignored. The heat radiates outside atmospheric transparency window and parasitically absorbs heat radiation from the atmosphere. Under this assumption, the net cooling power density, P_net_, is given by:

1 $${P}_{net}={P}_{dev}-{P}_{amb}$$

where P_dev_ and P_amb_ denote the radiation power density of the device and the ambient atmosphere, respectively. P_dev_ is designable through modifying the emissivity of the device.

2 $${P}_{dev}={\int }_{0}^{\pi /2}\pi \,\sin \,2\theta \,d\theta {\int }_{0}^{\infty }{U}_{B}({T}_{dev},\lambda ){\varepsilon }_{dev}({T}_{dev},\lambda ,\theta )d\lambda $$

where U_B_ is the spectral radiance of a blackbody which can be calculated from the Planck’s law. ε_dev_ is the emissivity of the cooling device. According to Kirchhoff’s law of thermal radiation, the absorptivity is equal to the emissivity. The angle-dependent emissivity of the device can be obtained by simulating the angle-dependent absorption using RCWA.

**Figure 3:**
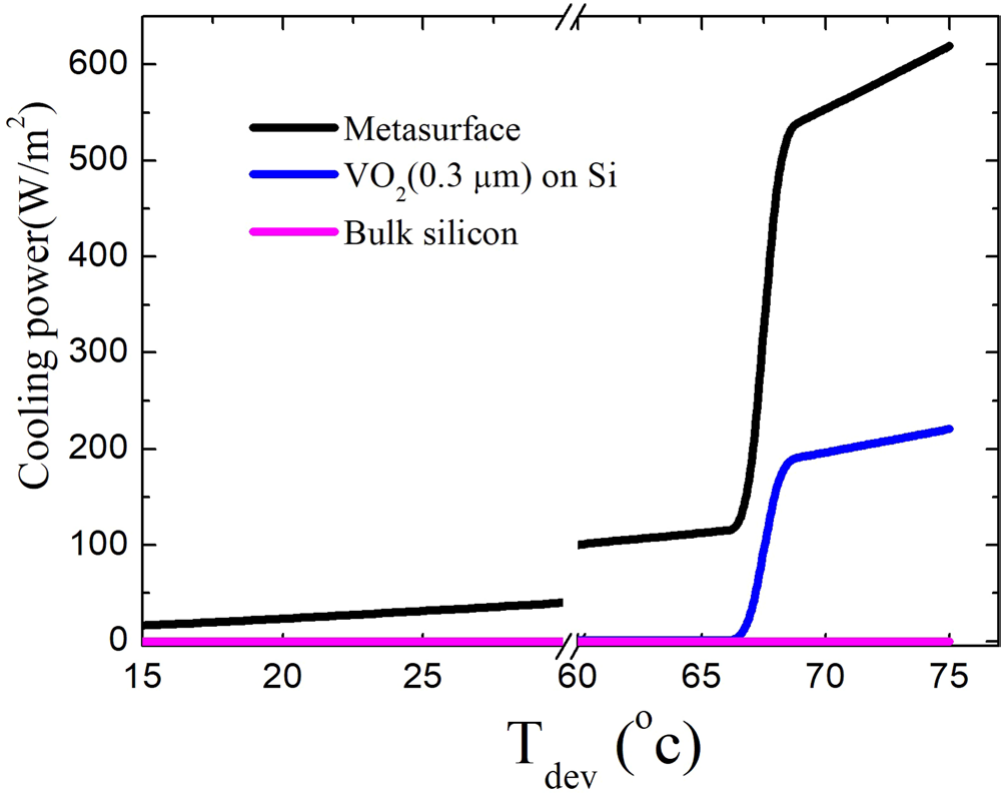
Theoretical net cooling power of the proposed VO_2_ metasurface (black), silicon substrate coated with 300 nm VO_2_ film (blue) and silicon substrate (pink) and silicon substrate as a function of the difference between the device and ambient temperature. The ambient temperature is assumed to be 27 °C.

The net cooling power density of the phase change metasurface as a function of the device temperature (T_dev_) is shown as the black solid line in Fig. 3. The ambient temperature is assumed to be 27 °C. The emissivity of the metasurface as a function of wavelength and observation angle is simulated using RCWA for both TE and TM-polarization. The geometric parameters of the VO_2_ metasurface are: W = 1800 nm, F_SiO2_ = 100 nm, F_VO2_ = 100 nm, T_SiO2_ = 400 nm, T_VO2_ = 400 nm and Λ_g_ = 3000 nm. As previously mentioned, we assume that the heat transfer is based on thermal radiation only. The emissivity of the atmosphere is taken from ref.^22^. For comparison, the net cooling power of both a bulk silicon and a silicon substrate coated with 300 nm VO_2_ film are calculated and shown as pink and blue solid line in Fig. 3, respectively. As shown in Fig. 3, the net cooling power slightly increases as the device temperature is below the phase change temperature of VO_2_. Once the device temperature is above the phase change temperature of VO_2_ the cooling power of the metasurface dramatically increases. Before and after VO_2_ phase change, the cooling power is 118 W/m^2^ W/m^2^ and 528 W/m^2^, as T_dev_ = 67 °C and T_dev_ = 69 °C, respectively. As a comparison, the net cooling power of silicon substrate coated with 300 nm VO_2_ film is 1.3 W/m^2^ and 187 W/m^2^ as T_dev_ = 67 °C and T_dev_ = 69 °C, respectively.

### Passive Temperature Control Based on VO_2_ Metasurface

Besides the radiative cooling effect, in this section, we will demonstrate how the proposed structure can be utilized as a passive temperature controller. Now, we assume that the metasurface is bonded on a heat source, for example, an electronic chip. At this time, net cooling power density becomes:

3 $${P}_{net}^{\text{'}}={P}_{dev}-{P}_{amb}-{P}_{sou}$$

where P_sou_ is the heat being transported to metasurface via heat conduction from a heat source. From the size of the metasurface and the specific heat capacity of SiO_2_ and VO_2_, we can simply calculate the required amount of heat needed to raise the temperature by ΔT Kelvin. Assuming an initial temperature, T_ini_, P_dev_(T_ini_, λ, θ) can be obtained via Eq. (2). Assuming a small derivative time interval Δt, we are able to calculate the device temperature of the metasurface after Δt, i.e. T_dev_(0 + Δt) where T_dev_(t = 0) = T_ini_. Then, P_dev_(T_dev_, λ, θ) can be obtained. Iteratively calculating T_dev_ (mΔt) for m = 1, 2, 3…, until T_dev_(mΔt) − T_dev_((m-1)Δt) approaches zero, T_dev_ as a function of time can be thus obtained.

**Figure 4:**
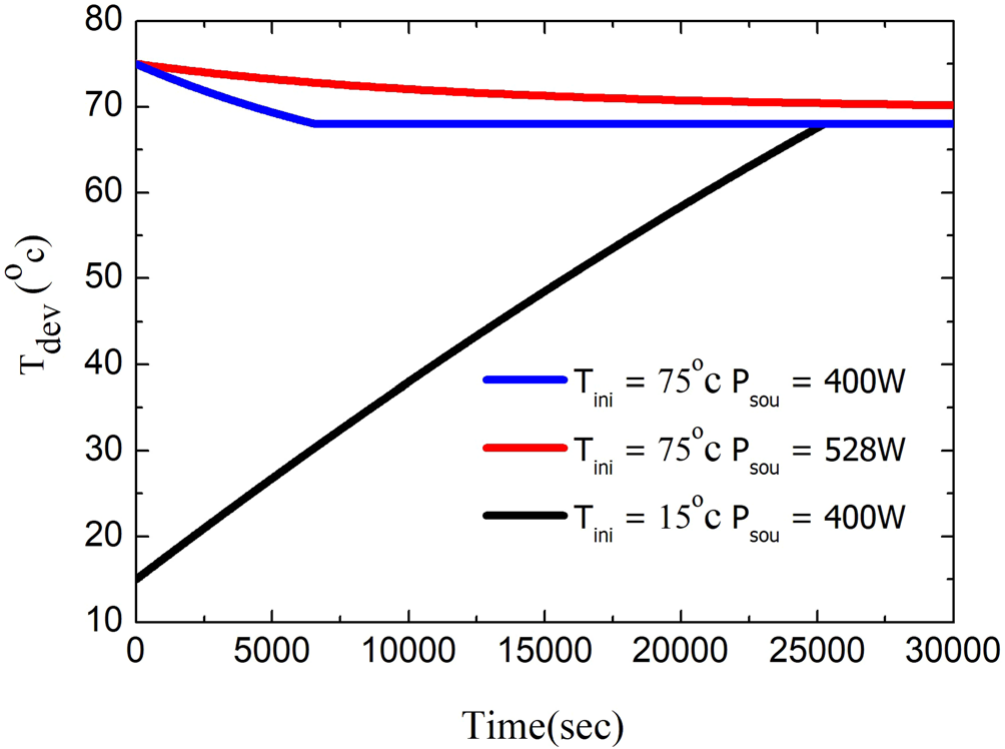
T_dev_ of the passive temperature control metasurface as a function of time. The ambient temperature is assumed to be 27 °C.

First, we consider the case for T_ini_ = 75 °C. Note that the P_net_ for just above and just below phase change temperature is 118 W/m^2^ and 528 W/m^2^, respectively. The T_dev_ as a function of time is shown in Fig. 4. For the red solid line, we assume P_sou_ = 528 W/m^2^, at this time, P’_net_ < 0 at 68^+ ^°C, where 68^+^ °C indicates the temperature is just above phase change temperature. Therefore, the final temperature cannot converge to a specific temperature. For the blue solid line, we assume P_sou_ = 400 W/m^2^, at this time, P’_net_ > 0 at 68^+^ °C and P’_net_ < 0 at 68^-^ °C where 68^-^ °C indicates the temperature is just below phase change temperature. Therefore, once T_dev_ > 68 °C, the device produces a radiative cooling effect. In contrast, once T_dev_ < 68 °C, the device produces a heating effect. As a result, T_dev_ finally converges to 68 °C. Similarly, as we assume the T_ini_ = 15 °C and P_sou_ = 400 W/m^2^, the device produces a heating effect, and the final temperature converges to 68 °C as well. As long as 118 W/m^2^ < P_sou_ < 528 W/m^2^, the device temperature can converge to the phase change temperature of VO^2^.

**Figure 5:**
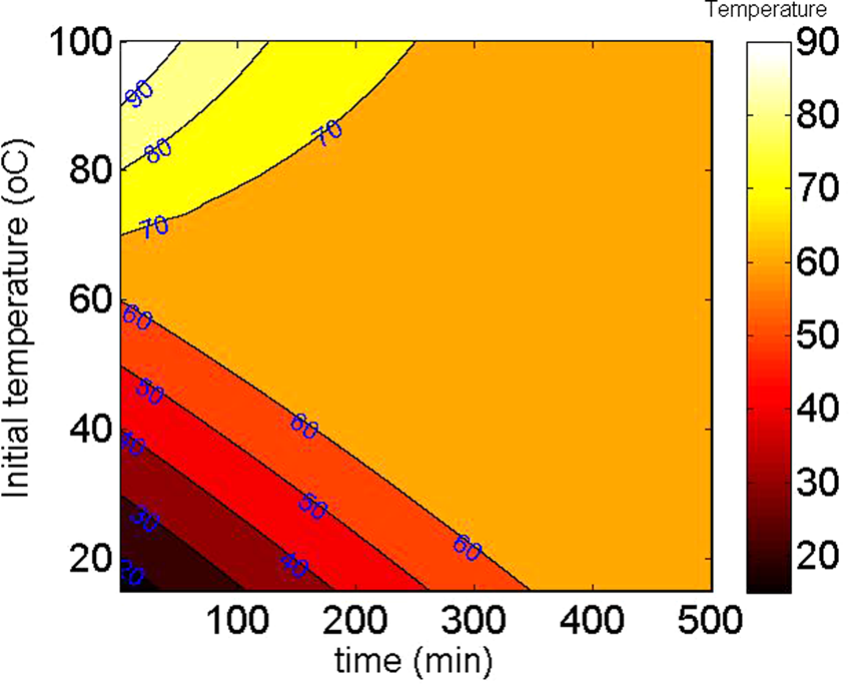
Metasurface temperature as a function of initial temperature and time for P_sou_ = 400 W/m^2^.

Finally, we demonstrate that the T_ini_ changes from 15 °C to 100 °C which is the common operation temperature of electronic chips. The T_sou_ is assumed to be 400 W/m^2^, which is also a common heat density generated from electronic chips. Figure 5 shows the device temperature as a function of Tini and time. It is shown that the final temperature can eventually converge to the corresponding phase change temperature. We, therefore, believe this passive temperature controller might be useful for maintaining the operation temperature of electronic devices. Although the control temperature can only be the phase change temperature of the utilized phase change material, it has been demonstrated that the phase change temperature of VO_2_ can be modified by doping. For example, the phase change temperature can be decreased by doping W into VO_2_ by 23 °C/wt%^23^. Alternatively, doping Mg or Mo can also significantly decrease the phase change temperature^24^. Consequently, we believe the controlled target temperature of the proposed passive temperature control metasurface can be modified by doping VO_2_.

## Conclusion

In summary, a mid-IR tunable metasurface consisting of a VO_2_ and SiO_2_ multilayer structure is proposed. It is shown that a FP-like fundamental mode at the transparency window of the atmosphere in VO_2_/SiO_2_/VO_2_ cavity can be thermally switched. The absorption spectrum of the proposed phase change metasurface can be dramatically changed at the transparent window as the device temperature goes below/above the phase change temperature of VO_2_. Theoretically, the radiative cooling power of the proposed metasurface can be thermally switched up to four-fold. Based on this huge cooling power switching ability, a passive temperature control application is theoretically demonstrated. We believe the proposed device can be applied for small radiative cooling and temperature control applications.

## Methods

The optical properties of the phase change metasurface are simulated using the RCWA method. It is a semi-analytical method for solving the transmission, reflection, diffraction and field profiles of a periodic structure. The structures and fields are decomposed as a sum of spatial harmonics during the simulation. Here, we use 23 harmonics to obtain convergent results due to the localized nature of the plasmonic resonances.
